# Luteolin Protects Chondrocytes from H_2_O_2_-Induced Oxidative Injury and Attenuates Osteoarthritis Progression by Activating AMPK-Nrf2 Signaling

**DOI:** 10.1155/2022/5635797

**Published:** 2022-02-01

**Authors:** Zhiqiang Zhou, Linlin Zhang, Yang Liu, Chaoming Huang, Wei Xia, Haibin Zhou, Zhengyu Zhou, Xiaozhong Zhou

**Affiliations:** ^1^Department of Orthopedics, The Second Affiliated Hospital of Soochow University, Suzhou 215000, China; ^2^Department of Orthopedics, The First Affiliated Hospital of Soochow University, Suzhou 215000, China; ^3^Department of Pharmaceutics, College of Pharmaceutical Sciences, Soochow University, Suzhou 215000, China; ^4^Department of Orthopedics, The Affiliated Suqian First People's Hospital of Nanjing Medical University, Suqian 223800, China; ^5^Department of Pathology, The Second Affiliated Hospital of Soochow University, Suzhou 215000, China; ^6^Laboratory Animal Center, Medical College of Soochow University, Suzhou 215000, China

## Abstract

Osteoarthritis (OA) is a chronic degenerative disease featured by cartilage erosion and inflammation. Luteolin, a member of the flavonoid family, has been shown to exert anti-inflammatory and antioxidative activities. However, the potential biological effects and underlying mechanism of luteolin on chondrocytes and OA progression remain largely elusive. In this study, the potential effect and mechanism of luteolin on OA were investigated *in vitro* and *in vivo*. Our data revealed that luteolin inhibited H_2_O_2_-induced cell death, apoptosis, oxidative stress, programmed necrosis, and inflammatory mediator production in primary murine chondrocytes. In addition, luteolin could activate the AMPK and Nrf2 pathways, and AMPK serves as a positive upstream regulator of Nrf2. *In vivo* results demonstrated the therapeutic effects of luteolin on OA in the DMM mouse model. Collectively, our findings showed that luteolin might serve as a novel and effective treatment for OA and provided a new research direction for clinical OA therapies.

## 1. Introduction

Osteoarthritis (OA) is a chronic joint degenerative disease with no cure currently [1–3]. The progressive chondrocyte apoptosis and extracellular matrix (ECM) degradation are the major pathogenic features of OA. Accumulating evidence has revealed that some oxidative stimuli will induce chondrocytes to generate reactive oxygen species (ROS) and inflammatory cytokines [4]. Inflammatory mediators, such as IL-1*β* and TNF-*α*, could damage the imbalance of cartilage homeostasis and activate inflammation response, resulting in chondrocyte apoptosis. As a major type of ROS, hydrogen peroxide (H_2_O_2_) induces mitochondrial damage, lipid peroxidation, and DNA damage, leading to ECM degradation and chondrocyte apoptosis [5]. Therefore, research on the discovery of novel and effective antioxidants to protect chondrocytes from oxidative injury has received increasing attention regarding OA treatment.

It is well-established that nuclear factor (erythroid-derived 2)-like 2 (Nrf2) plays a pivotal role in regulating cellular antioxidant processes in various cells, including chondrocytes [6–9]. Under quiescent circumstances, Nrf2 is bound by its repressor protein, Kelch-like ECH-associated protein 1 (Keap1), in the cytosol. Once activated by ROS or other oxidative stresses, Nrf2 dissociates from Keap1 and enters the nucleus. After binding to antioxidant response elements (AREs) in gene promoters, Nrf2 promotes the transcription and expression of several antioxidant enzymes, such as hem oxygenase 1 (HO-1), NAD (P) H quinone oxidoreductase-1 (NQO1), and *γ*-glutamyl cysteine ligase catalytic subunit (GCLC) [10, 11]. Furthermore, researches have revealed that Nrf2 boost protects chondrocytes against oxidative injury and inflammation, as well as attenuates OA progression in animal models [12, 13].

AMP-activated kinase (AMPK), a highly conserved serine/threonine kinase, serves as a master central hub that regulates cellular energy and metabolism homeostasis by suppressing inflammation and oxidative injury [14, 15]. Notably, emerging evidence has revealed that boost of the AMPK signaling pathway could protect cells against oxidative injury by targeting Nrf2 signaling [16, 17], indicating crosstalk between the AMPK and Nrf2 signaling pathways. However, the underlying mechanism by which the AMPK/Nrf2 pathway protects chondrocytes against oxidative stress remains largely unknown.

In the past few decades, most studies have focused on the therapeutic potential of natural products for OA treatment. Among those natural compounds, luteolin has attracted growing attention. As an important member of the flavonoid family, luteolin is widely found in many plants, including green pepper, celery, carrots, cauliflower, and spinach [18, 19]. Studies have shown that luteolin exhibits a series of therapeutic properties, such as anti-inflammatory, antioxidant, and antitumor effects [20, 21]. For example, Xiao et al. demonstrated that luteolin could inhibit cardiac ischaemia/reperfusion injury by activating the Nrf2 signaling pathway [18]. Furthermore, another study revealed that luteolin could activate the AMPK cascade to protect human umbilical vein endothelial cells from oxidative injury [19]. In addition, Wang et al. reported that luteolin alleviated allergic asthma by activating PI3K/Akt/mTOR signaling [22]. However, the potential biological effects and underlying mechanism of luteolin on chondrocytes and OA models remain largely elusive. Here, we explored the chondroprotective effects and the potential mechanism of luteolin in H_2_O_2_-induced chondrocytes and explored the *in vivo* protective effect of luteolin in the mouse OA model. The aim of the present study was to examine whether luteolin could protect primary murine chondrocytes from H_2_O_2_-induced injury and explore the potential mechanism. Additionally, the effect of luteolin on OA progression was assessed via the DMM mouse model.

## 2. Materials and Methods

### 2.1. Chemicals, Reagents, and Antibodies

**Figure 1:**
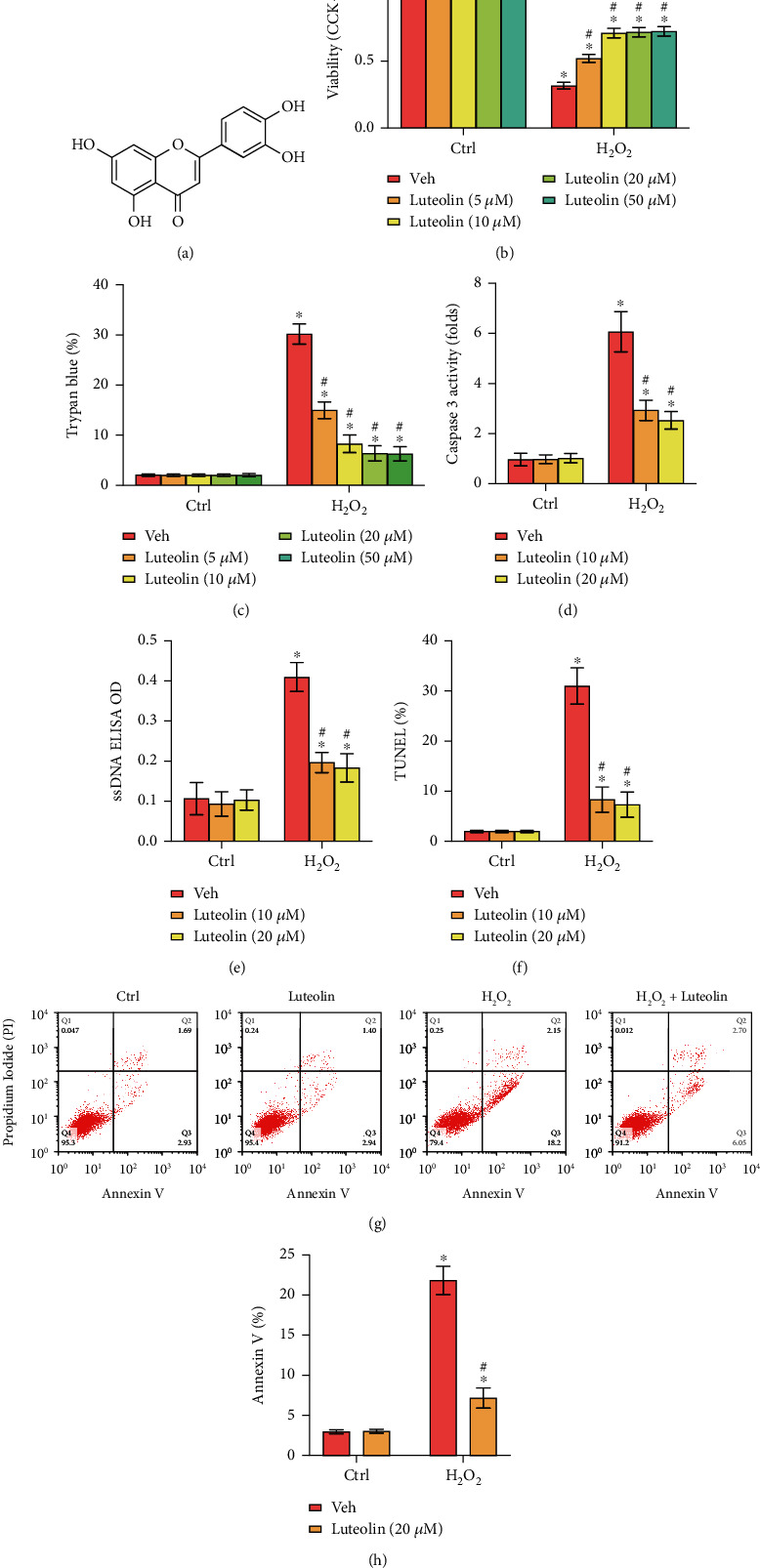
Luteolin inhibited H_2_O_2_-induced cell death and apoptosis in primary murine chondrocytes. (a) The chemical structure of luteolin. Primary murine chondrocytes (b–h) were treated for 2 h with the indicated concentration of luteolin or vehicle control (0.2% DMSO, Veh), followed by H_2_O_2_ (300 *μ*M) stimulation for 4 h. Cell viability (b), cell death (c), caspase-3 activity (d), the accumulation of ssDNA (e), apoptosis (f), and Annexin V staining (g, h) were tested by the corresponding assays. The data are expressed as the mean ± SD. ^∗^*P* < 0.05 vs. the Ctrl group. “Ctrl” indicates untreated control cells (the same for all figures). ^#^*P* < 0.05 vs. cells stimulated with H_2_O_2_ only.

Luteolin (purity > 98%, ab-120662, Figure 1(a)) was obtained from Abcam (Cambridge, UK). All antibodies were provided by Cell Signaling Technology (Shanghai, China) and Santa Cruz Biotech (Santa Cruz, CA). Carboxy-2′,7′-dichlorodihydrofluorescein diacetate (carboxy-H2DCFDA) fluorescent dye, TUNEL apoptosis detection kit (FITC-labelled), JC-1 dye, TRIzol reagent, and Lipofectamine 2000 were obtained from Invitrogen (Shanghai, China) and ThermoFisher Scientific (San Jose, CA). Puromycin, polybrene, the Annexin V-fluorescent-activated cell sorting (FACS) assay kit, the caspase-3 assay kit, and cell culture reagents were provided by Sigma-Aldrich (St. Louis, MO, USA).

### 2.2. Primary Murine Chondrocyte Culture

Ten C57BL/6 mice (less than 2 weeks of age) were euthanized by pentobarbital sodium, followed by harvest of articular cartilage pieces from the knee joints under sterile conditions. The cartilage was dissected, rinsed with phosphate-buffered saline PBS, and treated with 0.2% collagenase II at 37°C for 4 to 6 h. The digested cartilage tissue was centrifuged, and the cartilage tissue suspension was seeded in culture medium containing 10% FBS, DMEM/F12, and 1% penicillin/streptomycin under 5% CO_2_ at 37°C. The cells were harvested with 0.25% trypsin-ethylenediaminetetraacetic acid (EDTA) when they reached 80%~90% confluence, and only cells at passages 1 to 3 were used in our experiments.

### 2.3. Cell Viability

Primary murine chondrocytes (3000 cells per well) were seeded in 96-well plates. After the indicated treatments, cell viability was examined by the Cell Counting Kit-8 (CCK-8, Dojindo Co., Kumamoto, Japan) assay, and CCK-8 OD values at 450 nm were recorded.

### 2.4. Western Blotting

The detailed western blotting procedures were described elsewhere [23]. Antibodies were all used at 1 : 1,000 dilution unless otherwise indicated.

### 2.5. Quantitative Real-Time Polymerase Chain Reaction (qPCR)

After the indicated treatment, total cellular RNA was extracted with TRIzol reagent (Invitrogen, Shanghai, China), which was reverse transcribed using the ReverTra Ace qPCR RT kit (Toyobo, Tokyo, Japan) on an ABI Prism 7600H fast Real-Time PCR system (Applied Biosystems, Foster City, CA). The mRNA primers of the target genes were provided by Dr. Di [24]. Melt curve analysis was performed to calculate the product melting temperatures. The 2^−*ΔΔC*t^ method was used to quantify the targeted mRNAs, using *GAPDH* as the reference gene.

### 2.6. Mitochondrial Immunoprecipitation (Mito-IP)

For each treatment, mitochondria were isolated from cultured chondrocytes with a Mitochondria Isolation Kit for Cultured Cells from Thermo Scientific (Hudson, NH) and then lysed with lysis buffer (20 mM Tris, pH 7.4, 135 mM NaCl, 1.5 mM MgCl_2_, 1 mM EGTA, 10% glycerol, and 1% Triton X-100). The lysates (400 *μ*g of each sample) were precleared and incubated with anti-Cyp-D antibodies (Santa Cruz Biotech, Santa Cruz, CA, USA). The mitochondrial complexes were then captured with protein G Sepharose beads (Sigma). Cyclophilin-D- (Cyp-D-) p53-adenine nucleotide translocator-1 (ANT-1) associations were examined by western blotting.

### 2.7. Nrf2 shRNA and AMPK*α*1 shRNA

Nrf2 shRNA and AMPK*α*1 shRNA lentiviral particles (Santa Cruz Biotech, Santa Cruz, CA) were individually added to cultured primary murine chondrocytes for 24 h, followed by puromycin selection for 12 days. In stable cells, over 95% knockdown of target proteins (Nrf2 and AMPK*α*1) was detected by western blotting and qPCR analyses. Control cells were transduced with lentiviral scramble control shRNA (sh-C).

### 2.8. Nrf2 KO and AMPK*α*1 KO by CRISPR/Cas9

The lenti-CRISPR-GFP-Nrf2/AMPK*α*1 KO constructs were individually transfected into primary murine chondrocytes with Lipofectamine 2000. GFP-positive cells were sorted FACS. Single cells were further cultured in 96-well plates to generate monoclonal cells, followed by puromycin selection of stable cells. Nrf2 KO and AMPK*α*1 KO were verified by western blotting and qPCR analyses.

### 2.9. Mitochondrial Depolarization

In the presence of mitochondrial depolarization, JC-1 fluorescent dye aggregates in mitochondria to form green monomers [25]. The method for the JC-1 assay was reported previously [26].

### 2.10. Coimmunoprecipitation (Co-IP)

The methods for Co-IP were reported previously [27]. In brief, 800 *μ*g of total cell lysates were precleared with protein A/G Sepharose (“Beads”, Sigma-Aldrich). Then, the anti-Keap1 antibody was added to the lysates and incubated overnight, followed by the protein A/G Sepharose adding to the lysates. The Keap1-immunoprecipitated proteins were then captured by the beads and examined by western blotting.

### 2.11. Intracellular ROS Measurement

ROS levels were measured by the carboxy-H2DCFDA dye assay according to the previous report [28].

### 2.12. Lipid Peroxidation

As reported [28], the level of cellular lipid peroxidation was quantified by thiobarbituric acid reactive substance (TBAR) activities.

### 2.13. Superoxide Detection

A superoxide colorimetric assay kit (BioVision, San Francisco, CA) was used to measure cellular superoxide levels based on the kit protocol.

### 2.14. TUNEL Assay

Cells were initially seeded in six-well tissue culture plates (30,000 cells/cm^2^). Following the treatments, the cells were stained with TUNEL (5 mM) for 30 min at room temperature in the dark. Cells with positive nuclear TUNEL staining were labelled apoptotic cells. The TUNEL ratio (TUNEL/DAPI × 100%) was calculated by counting 500 cells in five random fields of views (1 × 200 magnification) for each treatment.

### 2.15. Glutathione Levels

Primary murine chondrocytes were seeded in six-well tissue plates. Following H_2_O_2_ stimulation, the levels of GSH and GSSG were tested, and the GSH/GSSG ratio was determined using a previously described protocol [29].

### 2.16. Caspase-3 Activity

Primary murine chondrocytes were seeded in six-well tissue plates. Following H_2_O_2_ stimulation, 30 *μ*g of total cell lysates were incubated with AFC-bound caspase-3 substrate (Invitrogen, Shanghai, China). The AFC absorbance, which indicates the relative caspase-3 activity, was measured by a Fluoroskan Ascent FL instrument at excitation and emission wavelengths of 355 nm and 525 nm, respectively.

### 2.17. Annexin V FACS

After being treated, the cells were incubated with Annexin V and propidium iodide (PI) (both 5 mg/mL). Then, FACS was performed on a FACSCalibur (BD Biosciences, Shanghai, China) to analyze the cells. The Annexin V ratio was recorded.

### 2.18. Single-Stranded DNA (ssDNA)

The cellular ssDNA level was tested following the previously reported method [26].

### 2.19. ARE Promoter Activity

Chondrocytes were seeded in six-well plates and transfected with pGL4.37 and pGL4.74 plasmids in accordance with the manufacturer's protocol. After the indicated treatments, a dual-luciferase reporter assay system (Dual-Glo® Luciferase Assay System) was used to analyze ARE-driven promoter activity.

### 2.20. Measurement of NO, PGE_2_, TNF-*α*, IL-6, Collagen II, Aggrecan, ADAMTS-5, and MMP13

The NO concentration in culture medium was measured by Griess reagent as previously described [30]. The concentrations of PGE_2_, TNF-*α*, IL-6, collagen II, aggrecan, ADAMTS-5, and MMP13 in cell culture supernatants were measured by ELISA kits (R&D Systems, Minneapolis, MN) based on the manufacturer's instructions.

### 2.21. Animal Model

Male C57BL/6 mice (20-25 g; 8 weeks old) were obtained from Shanghai Animal Centre of the Chinese Academy of Sciences and housed under specific pathogen-free (SPF) conditions in our facility. All animal procedures were conducted in accordance with the National Institutes of Health guide for the care and use of Laboratory animals (NIH Publications No. 8023, revised 1978) and approved by the Institutional Animal Care and Use Committee of Soochow University. The mouse OA model was established by surgical destabilization of the medial meniscus (DMM) of the right knee as described previously [31]. Then, the mice were randomly assigned to three groups: sham group (*n* = 10), DMM group (OA group, *n* = 10), and DMM+luteolin group (*n* = 10).

### 2.22. Histopathological Analysis

Freshly dissected mouse knee joints were fixed in 4% paraformaldehyde (PFA) for 24 h and decalcified in a 10% EDTA solution for 4 weeks. The knee samples were embedded in paraffin blocks and sliced (5 *μ*m). After a series of processing steps, the joint sections were stained with hematoxylin and eosin (HE) and safranin O/fast green (Sigma-Aldrich, Oakville, Ontario, Canada) in accordance with the manufacturer's recommendations. The slides were assessed by two independent histology researchers in a blinded manner. The Osteoarthritis Research Society International (OARSI) scoring system was used to quantify the degeneration of cartilage as previously described [32].

### 2.23. Statistical Analysis

Each experiment was repeated in triplicate. The data are presented as the mean ± standard deviation (SD). Statistical analysis was conducted through SPSS 20.0. Statistical analyses were performed by one-way analysis of variance (ANOVA), and the Tukey test was used for comparisons between groups. OARSI scores were analyzed by the Kruskal–Wallis *H* test. *P* < 0.05 was considered statistically significant.

## 3. Results

### 3.1. Luteolin Inhibited H_2_O_2_-Induced Cell Death and Apoptosis in Primary Murine Chondrocytes

The effect of luteolin on cell death and apoptosis was evaluated. The results demonstrated that H_2_O_2_ induced robust cell viability reductions (Figure 1(b)) and cell death (Figure 1(c)), which were distinctly reversed by luteolin treatment. In addition, H_2_O_2_ induced an increase in caspase-3 activity (Figure 1(d)), ssDNA accumulation (Figure 1(e)), and the upregulation of the TUNEL-positive nuclei ratio (Figure 1(f)) and Annexin V-positive ratio (Figures 1(g) and 1(h)), indicating apoptosis activation. Conversely, these effects were dramatically ameliorated by luteolin treatment (Figures 1(d)–1(h)). Notably, treatment with luteolin alone failed to affect chondrocyte viability or cell death (Figures 1(d)–1(h)). These results suggested that luteolin treatment effectively protected chondrocytes from H_2_O_2_-elicited cell death and apoptosis.

### 3.2. Luteolin Inhibited H_2_O_2_-Stimulated Oxidative Injury and Programmed Necrosis in Primary Murine Chondrocytes

**Figure 2:**
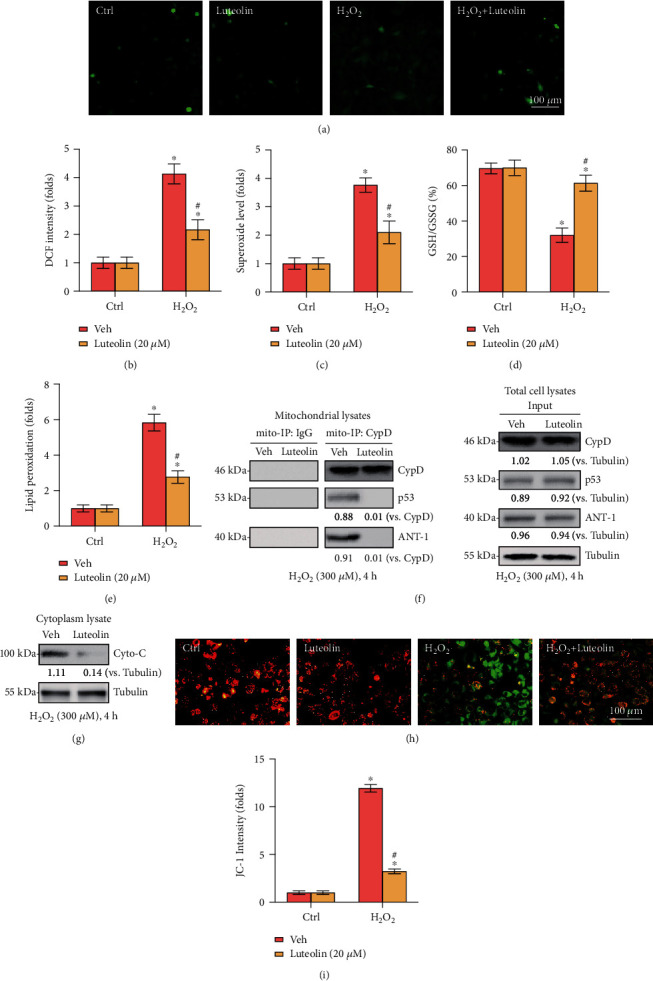
Luteolin inhibited H_2_O_2_-stimulated oxidative injury and programmed necrosis in primary murine chondrocytes. Primary murine chondrocytes (a–i) were treated with luteolin (20 *μ*M) or vehicle control (0.2% DMSO, Veh) for 2 h, followed by H_2_O_2_ (300 *μ*M) stimulation for 4 h. ROS production (a, b), superoxide levels (c), GSH/GSSG ratios (d), and lipid peroxidation (e) were examined by the corresponding assays. Mitochondrial CypD-p53-ANT-1 associations (Mito-IP, (f)), Cyto-C release (g), and mitochondrial depolarization (h, i) were tested. Error bars represent the mean ± SD. ^∗^*P* < 0.05 vs. the Ctrl group. ^#^*P* < 0.05 vs. cells stimulated with H_2_O_2_ only.

The effect of luteolin on H_2_O_2_-stimulated ROS generation and oxidative injury in chondrocytes was evaluated. Our results showed that ROS production (DCF-DA intensity increase, Figures 2(a) and 2(b)) was potently increased in response to H_2_O_2_ stimulation, and oxidative injury was significantly induced, which was supported by superoxide accumulation (Figure 2(c)), a reduction in the reduced glutathione/oxidized disulfide form glutathione (GSH/GSSG) ratio (Figure 2(d)), and the presence of lipid peroxidation (TBAR intensity increase, Figure 2(e)). Notably, luteolin treatment significantly reversed these effects (Figures 2(a)–2(e)).

Additionally, programmed necrosis was also activated in H_2_O_2_-stimulated primary murine chondrocytes, as indicated by the association of mitochondrial CypD-p53-ANT-1 (Figure 2(f)), cytosolic cytochrome C (Cyto-C) release (Figure 2(g)), and mitochondrial depolarization (Figures 2(h) and 2(i)). Again, these effects were largely attenuated by luteolin treatment. Collectively, these data suggested that luteolin reversed H_2_O_2_-stimulated oxidative injury and programmed necrosis in murine chondrocytes.

### 3.3. Luteolin Inhibited H_2_O_2_-Induced Inflammatory Mediator Production and Ameliorated H_2_O_2_-Induced ECM Degradation

**Figure 3:**
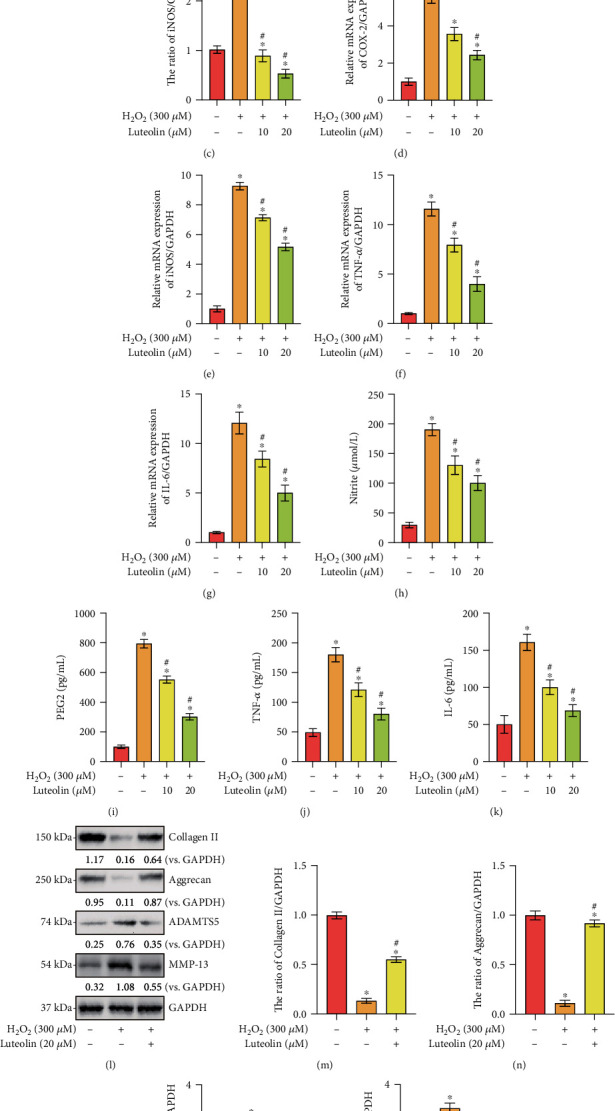
Luteolin inhibits H_2_O_2_-induced inflammatory mediators and ameliorated H_2_O_2_-induced ECM degradation. Primary murine chondrocytes (a–p) were treated for 2 h with the indicated concentration of luteolin or vehicle control (0.2% DMSO, Veh), followed by H_2_O_2_ (300 *μ*M) stimulation for 4 h. (a) The protein levels of iNOS and COX-2 in chondrocytes. (e–h) The mRNA expressions of iNOS, COX-2, TNF-*α*, and IL-6. (c, d) Quantitative western blot analysis of iNOS and COX-2 in chondrocytes. (i–l) The levels of NO, PGE2, TNF-*α*, and IL-6 in cell supernatants were analyzed by ELISA. The protein (b) and mRNA (m–p) expression levels of collagen II, aggrecan, ADAMTS5, and MMP-13 in chondrocytes were determined. The data are expressed as the mean ± SD. ^∗^*P* < 0.05 vs. the Ctrl group. ^#^*P* < 0.05 vs. cells stimulated with H_2_O_2_ only.

To examine the protective effect of luteolin against H_2_O_2_-induced inflammatory mediators and ECM degradation in chondrocytes, inflammatory mediators, including COX-2, iNOS, TNF-*α*, IL-6, nitric oxide (NO), and PGE2, and the expression of ECM components, including ADAMTS-5, MMP-13, collagen II and aggrecan, were examined. As presented in Figures 3(a)–3(g), H_2_O_2_-induced upregulations of iNOS, COX-2, TNF-*α*, and IL-6 were markedly inhibited by luteolin treatment. In addition, the increased levels of endogenous NO, PGE2, TNF-*α*, and IL-6 induced by H_2_O_2_ stimulation were dramatically blocked by luteolin (Figures 3(h)–3(k)). Furthermore, H_2_O_2_ markedly mitigated the levels of collagen II and aggrecan, which contributed to chondrocyte survival, and significantly enhanced the expression of MMP-13 and ADAMTS-5, which were the major ECM degradation enzymes, and all these H_2_O_2_-induced alterations were reversed by luteolin treatment (Figures 3(l)–3(p)). Taken together, these data suggested that luteolin inhibited H_2_O_2_-induced inflammatory mediators and protects chondrocytes against ECM degradation.

### 3.4. Luteolin Activated AMPK and Nrf2 Signaling in Primary Murine Chondrocytes

**Figure 4:**
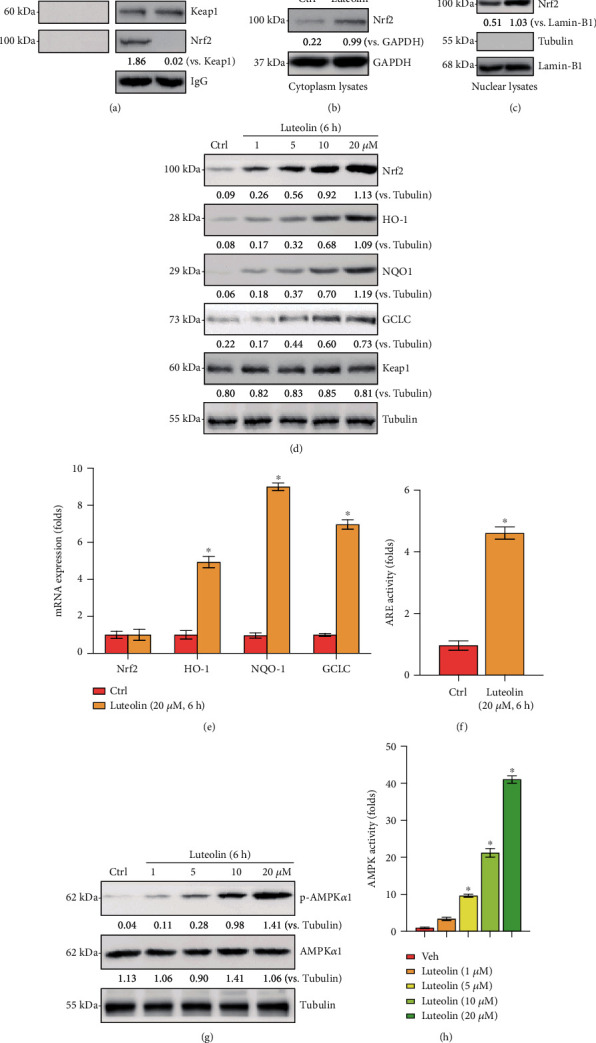
Luteolin activated AMPK and Nrf2 signaling in primary murine chondrocytes. Primary murine chondrocytes were treated with the indicated concentration of luteolin or vehicle control (0.2% DMSO, Veh). The Keap1/Nrf2 interaction was examined by coimmunoprecipitation (Co-IP) (a). The expression of the indicated proteins was examined by western blotting (b, c). Protein (d, g) and mRNA (e) expressions of the indicated proteins in total cell lysates are presented. ARE and AMPK activities were examined (f, h). The data are presented as the mean ± SD. ^∗^*P* < 0.05 vs. the Ctrl group.

To further explore the protective mechanism of luteolin treatment on H_2_O_2_-induced oxidative injury in chondrocytes, the AMPK and Nrf2 pathways were analyzed by western blotting. As demonstrated in Figures 4(a) and 4(b), the coimmunoprecipitation assay results revealed that Keap1 immunoprecipitated with Nrf2 and that treatment with luteolin damaged the connection between Keap1 and Nrf2, resulting in the stabilization and accumulation of the Nrf2 protein in the cytoplasm, while Keap1 expression remained unchanged. In addition, elevated Nrf2 protein levels were detected in the nuclear fractions of luteolin-treated chondrocytes (Figure 4(c)), indicating the translocation of stabilized Nrf2 from the cytosol to the nucleus. Importantly, luteolin robustly strengthened ARE luciferase activity in primary murine chondrocytes (Figure 4(f)), leading to enhanced transcription of Nrf2-dependent genes (HO-1, NQO1, and GCLC; Figures 4(d) and 4(e)). Notably, the Nrf2 protein level was robustly increased in luteolin-treated chondrocytes, whereas the Nrf2 mRNA level remained unchanged (Figure 4(e)). The results indicated that luteolin treatment resulted in the stabilization, nuclear translocation, and activation of Nrf2 in primary murine chondrocytes.

Next, we examined AMPK signaling. As shown in Figure 4(g), luteolin upregulated the phosphorylation levels of AMPK*α*1 in primary murine chondrocytes, while the total levels of AMPK*α*1 remained unchanged. Additionally, AMPK activity was potently augmented by luteolin treatment (Figure 4(h)). Thus, luteolin activated AMPK signaling in primary murine chondrocytes. Collectively, these data showed that luteolin-induced protection against H_2_O_2_ in chondrocytes might occur through the AMPK/Nrf2 pathway.

### 3.5. Nrf2 Signaling Activation Mediated Luteolin-Induced Chondroprotection from H_2_O_2_

**Figure 5:**
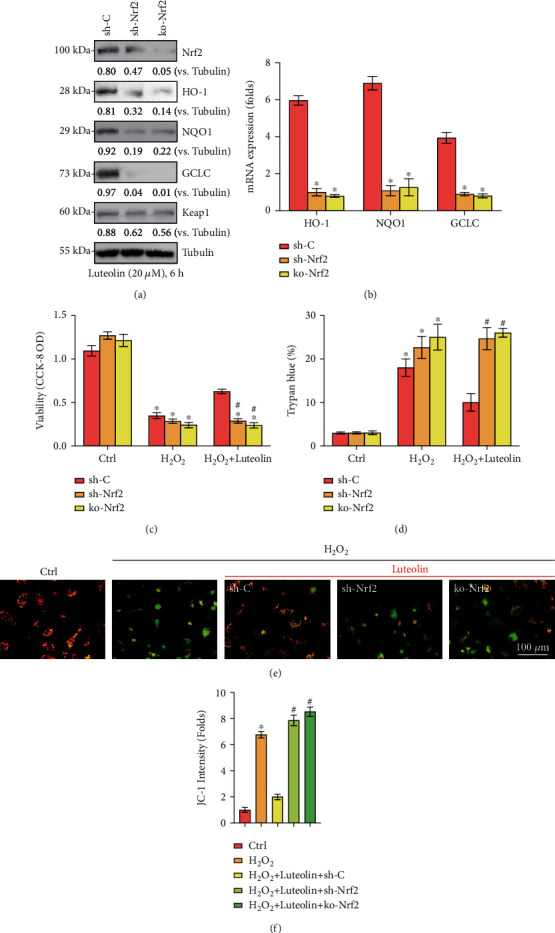
Nrf2 signaling activation mediated luteolin-induced cytoprotection from H_2_O_2_. Stable primary murine chondrocytes with the indicated Nrf2 shRNA (sh-Nrf2) or the CRISPR/Cas9-Nrf2-KO-GFP construct (ko-Nrf2), as well as control cells with scramble control shRNA (sh-C), were established and cultured, and the expression of the indicated genes was measured (a, b). Cells were treated for 2 h with luteolin (20 *μ*M), followed by H_2_O_2_ (300 *μ*M) stimulation for the indicated times. Cell viability (c) and apoptosis (d) were measured by CCK-8 and Trypan blue assays, respectively. (e) Mitochondrial depolarization was tested. Quantified values are the mean ± SD. ^∗^*P* < 0.05 vs. the Ctrl group. ^#^*P* < 0.05 vs. “sh-C” cells.

Next, the relationship between the Nrf2 cascade and luteolin-induced cellular protection in H_2_O_2_-stimulated chondrocytes was further examined. Chondrocytes were transfected with lentiviral Nrf2 shRNA, and stable cells were selected by puromycin. Besides, a lentiCRISPR/Cas9-Nrf2-GFP-knockout (KO) construct was used to knock out Nrf2 in primary murine chondrocytes, and stable cells were established. Our findings demonstrated that Nrf2 protein expression was dramatically suppressed in Nrf2 shRNA and Nrf2-KO chondrocytes treated with luteolin (Figure 5(a)). Furthermore, luteolin-stimulated Nrf2 protein stabilization, as well as enhancement of HO-1, NQO1, and GCLC, was abolished in Nrf2 shRNA and KO cells (Figures 5(a) and 5(b)). More importantly, H_2_O_2_-induced cell viability decrease (Figure 5(c)), cell death (Figure 5(d)), and mitochondrial depolarization (Figures 5(e) and 5(f)) were intensified in Nrf2-silenced cells, and luteolin treatment did not eliminate these effects (Figures 5(c) and 5(d)). Therefore, Nrf2 silencing or depletion affects luteolin-induced effects on primary murine chondrocytes. Taken together, our data showed that Nrf2 cascade activation mediated the protective effect of luteolin against H_2_O_2_ in chondrocytes.

### 3.6. AMPK Activation Mediated Luteolin-Induced Chondroprotection against H_2_O_2_

**Figure 6:**
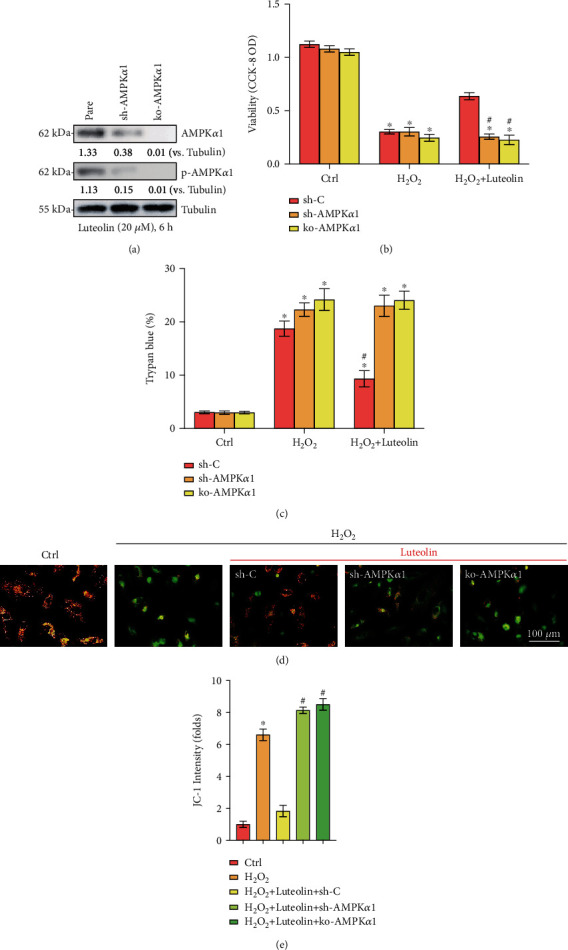
AMPK activation mediated luteolin-induced cytoprotection against H_2_O_2_. Stable primary murine chondrocytes with the indicated AMPK*α*1 shRNA (sh-AMPK*α*1) or the CRISPR/Cas9-Nrf2-KO-GFP construct (ko-AMPK*α*1), as well as control cells with scramble control shRNA (sh-C), were established and cultured, and the expression of the indicated genes was examined (a). Cells were treated for 2 h with luteolin (20 *μ*M), followed by H_2_O_2_ (300 *μ*M) stimulation for the indicated times. Cell viability (b) and apoptosis (c) were measured by CCK-8 and Trypan blue assays, respectively. (d, e) Mitochondrial depolarization was tested. Quantified values are the mean ± SD. ^∗^*P* < 0.05 vs. the Ctrl group. ^#^*P* < 0.05 vs. “sh-C” cells.

To explore whether AMPK signaling activation mediates the luteolin-induced protection against H_2_O_2_, genetic methods were employed to silence AMPK activation. First, AMPK*α*1 shRNA lentivirus was transfected into primary murine chondrocytes, followed by the establishment of stable cells via puromycin selection (sh-AMPK*α*1). In addition, a lentiCRISPR/Cas9-AMPK*α*1-GFP-KO construct was used to knock out AMPK*α*1 in primary murine chondrocytes, and stable cells were established (ko-AMPK*α*1). In sh-AMPK*α*1 and ko-AMPK*α*1 cells, reduced AMPK*α*1 expression levels were confirmed by western blotting (Figure 6(a)). More importantly, cell viability reductions (Figure 6(b)), cell death (Figure 6(c)), and mitochondrial depolarization (JC-1 intensity, Figures 6(d) and 6(f)) induced by H_2_O_2_ were not affected by luteolin treatment after AMPK*α*1 silencing or depletion. These data indicated that AMPK activation mediated luteolin-induced protection against H_2_O_2_ in chondrocytes.

### 3.7. AMPK of Downstream Nrf2 Signaling Activation Was Required for Luteolin-Induced Chondroprotection from H_2_O_2_

**Figure 7:**
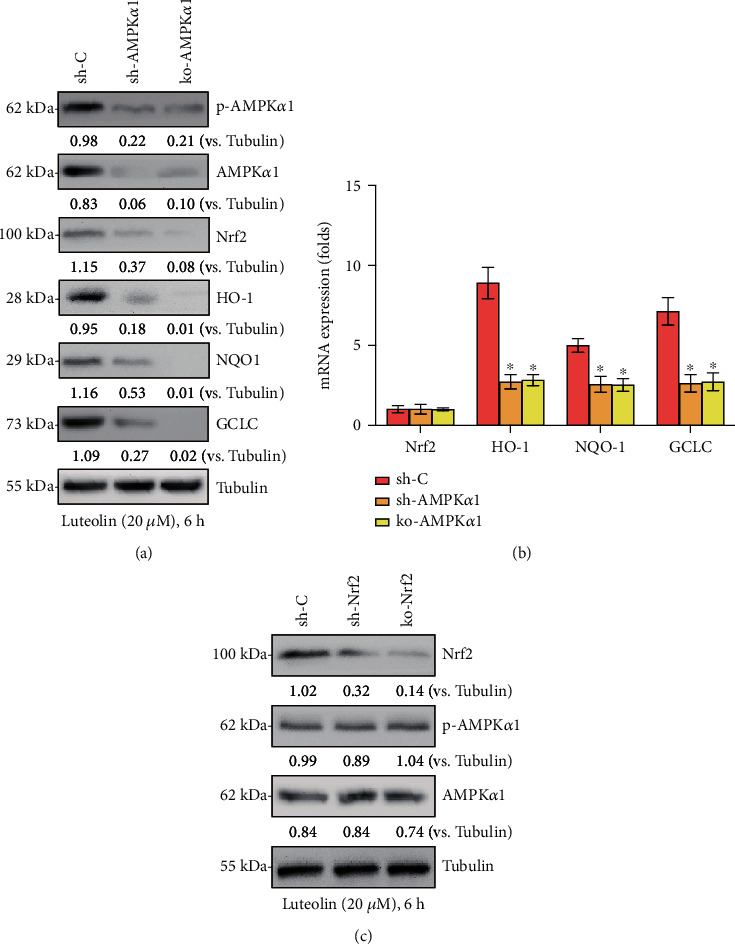
AMPK downstream Nrf2 signaling activation was required for luteolin-induced cytoprotection from H_2_O_2_. Stable primary murine chondrocytes with AMPK*α*1 shRNA (“sh-AMPK*α*1”) and CRISPR/Cas-9 AMPK*α*1-KO construct (“ko-AMPK*α*1”), as well as control cells with scramble control shRNA (sh-C), were treated with luteolin (20 *μ*M) for 2 h, followed by H_2_O_2_ stimulation for 4 h. The protein and mRNA expressions of the indicated genes were presented (a, b). Stable primary murine chondrocytes with Nrf2 shRNA (“sh-Nrf2” cells) and CRISPR/Cas-9 Nrf2-KO constructs (“ko-Nrf2” cells), as well as control cells with scramble control shRNA (sh-C), were treated with luteolin (20 *μ*M) for 2 h, followed by H_2_O_2_ stimulation for 48 h. The expressions of the indicated proteins were shown (c). The data are the mean ± SD. ^∗^*P* < 0.05 vs. “sh-C” cells (a, b).

Based on the widely reported interaction between the Nrf2 and AMPK cascades, the association of luteolin-mediated AMPK and Nrf2 activation was further investigated in primary murine chondrocytes. Genetic strategies (shRNA and CRISPR/Cas9-KO) were used to block AMPK and Nrf2. The results showed that luteolin-mediated Nrf2 activation was effectively blocked by AMPK shRNA or AMPK KO (Figures 7(a) and 7(b)). In contrast, Nrf2 silencing or depletion had no significant effect on luteolin-induced AMPK activation (Figure 7(c)). These data suggested that luteolin-induced AMPK activation acted as an upstream signal for Nrf2 signaling activation.

### 3.8. Luteolin Ameliorated OA Progression in the DMM Mouse Model

**Figure 8:**
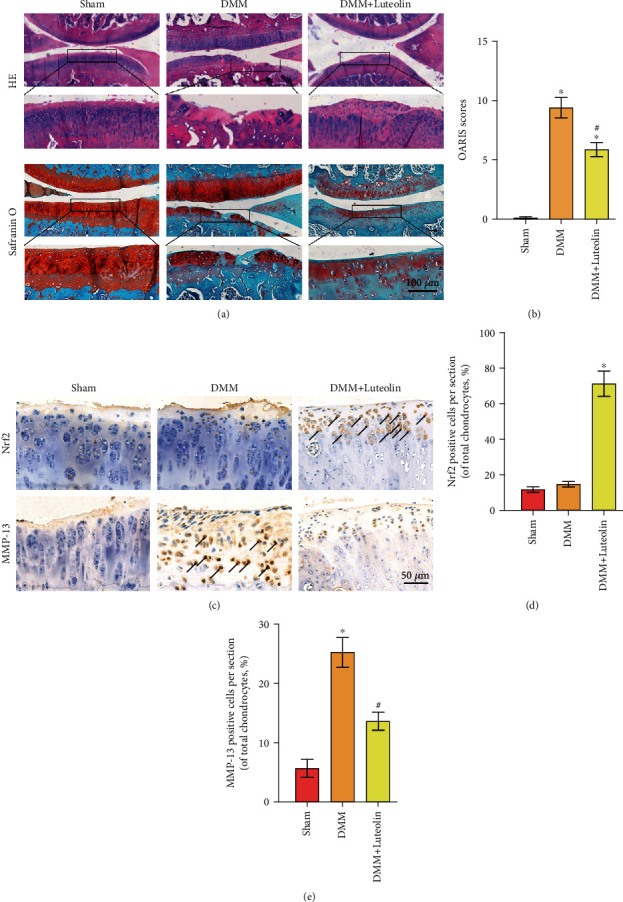
Luteolin ameliorated OA progression in the DMM mouse model. (a) Histological analysis of OA was assessed by HE staining and safranin O staining. (b) OARSI scores were calculated in the different groups. (c) The expressions of Nrf2 and MMP-13 in cartilage samples were examined by immunohistochemistry. (d, e) Quantitative analysis of Nrf2 positive expression in the sections. The values presented are the means ± SD. ^∗^*P* < 0.05 vs. the sham group. ^#^*P* < 0.05 vs. the DMM group.

To assess the protective effect of luteolin on OA progress *in vivo*, DMM was established in mice, followed by intragastric administration of 10 mg/kg/day luteolin in 0.5% carboxymethylcellulose (CMC) or vehicle alone (0.5% CMC) for 8 consecutive weeks until the mice were sacrificed. The protective effect of luteolin on cartilage morphological structure was examined. As presented by HE and safranin O staining (Figure 8(a)), the DMM group exhibited robust cartilage damage, chondrocyte decreases, and notable proteoglycan decreases compared to those in the sham control group. Moreover, these effects were mitigated in the luteolin-treated DMM group (DMM+luteolin), which revealed a smoother cartilage surface than that of the DMM group. OARSI scores were also determined for quantitative analysis (Figure 8(b)). The DMM group had significantly higher OARSI scores than the sham control group. Contrarily, the DMM+luteolin group showed markedly lower OARSI scores than the DMM group. Furthermore, to investigate the effect of luteolin on ECM *in vivo*, immunohistochemical staining of Nrf2 and MMP13 in cartilage samples was carried out. The immunohistochemistry results (Figures 8(c)–8(e)) demonstrated higher expression of MMP13 in the DMM group than in the sham group, and Nrf2 expression showed no significant difference between the sham and DMM groups; however, the level of MMP13 was decreased and Nrf2 was upregulated in the DMM+luteolin group. Collectively, these results indicated that luteolin alleviated OA progression *in vivo*.

## 4. Discussion

In the current study, we investigated the potential protective effect of luteolin on H_2_O_2_-induced chondrocytes and DMM-induced OA in mice. The results showed that luteolin distinctly mitigated H_2_O_2_-induced cell death and apoptosis in primary murine chondrocytes and markedly inhibited OA progression in the mouse model. Besides, our study revealed that the AMPK/Nrf2 signaling pathway was involved in luteolin-induced cytoprotection.

Growing evidence reveals that inflammation and oxidative stress are the major pathological processes responsible for OA progression [4, 5]. The excessive ROS could damage biomolecules or modify proteins and genes to activate signaling cascades [33]. As a result, some transcription factors and proinflammatory genes stimulated by ROS lead to the onset and development of inflammation [34]. Reflexively, an enhanced ROS generation due to the inflammatory response induces oxidative stress and tissue injury, such as ECM degradation. Thus, inflammation and oxidative stress are inextricably interrelated, creating a vicious cycle to provoke the occurrence and progression of a series of diseases, including OA.

Oxidative stress resulting from excessive ROS generation stimulates lipid peroxidation, protein damage, and DNA breaks, ultimately leading to chondrocyte apoptosis and articular cartilage degradation, ultimately contributing to OA pathogenesis [5]. In contrast, inhibiting ROS production and oxidative stress can effectively protect chondrocytes. In the current study, treatment with luteolin dramatically reversed the apoptosis-related effects of H_2_O_2_, as evidenced by ROS production, an increase in caspase-3 activity, and the accumulation of ssDNA. These results indicated that luteolin potently ameliorated H_2_O_2_-induced oxidative injury and apoptosis in chondrocytes.

Further, inflammatory factors have been shown to play a principal role in OA development. iNOS can synthesize NO, which not only stimulates MMP production but also inhibits collagen II and proteoglycan generation, ultimately leading to ECM degradation [35]. PGE2, another core inflammatory mediator, is produced by COX-2 and facilitates ECM degradation by increasing the levels of MMP-13 and ADAMTS5 [36]. Eventually, the accumulation of NO and PGE2 results in chondrocyte apoptosis and contributes to OA progression [37]. Collagen II and aggrecan, the main components of the ECM, are synthesized and secreted by chondrocytes and are usually degraded under inflammatory conditions by MMP-13 and ADAMTS5, respectively [38]. Thus, articular cartilage degeneration could be delayed by inhibiting the expression of inflammatory mediators. Our study showed that the H_2_O_2_-induced generation of PGE_2_, NO, TNF-*α*, IL-6, COX-2, and iNOS was reversed by luteolin in OA chondrocytes. Furthermore, luteolin suppressed the generation of MMP-13 and ADAMTS5, as well as the degradation of collagen II and aggrecan. These data suggested that luteolin could effectively inhibit the production of inflammatory cytokines and protect chondrocytes from H_2_O_2_.

Then, the underlying mechanism of luteolin-induced cytoprotection was investigated. It has been generally recognized that both the AMPK and Nrf2 signaling pathways play vital roles in regulating cellular energy and metabolism homeostasis by inhibiting inflammation and ROS generation, thus protecting cells under stress conditions. Therefore, the potential connection between luteolin-mediated protective effects and AMPK or Nrf2 signaling was examined.

AMPK is a phylogenetically conserved fuel-sensing enzyme and plays a vital role in regulating cellular energy homeostasis [14, 15]. It exists as a heterotrimer composed of a catalytic *α* subunit and regulatory *β* and *γ* subunits. The kinase is activated by stresses that inhibit ATP generation or promote its consumption, such as glucose deprivation, hypoxia, and ischemia. Recent studies have demonstrated that AMPK serves as a therapeutic target for various metabolic diseases. For instance, AMPK is reported to be involved in the regulation of nonalcoholic fatty liver disease [39], ischemic stroke-induced brain injury [40, 41], acute lung injury [42], and OA progression [43]. In addition, it is well-established that Keap1/Nrf2 system forms the cellular defense against oxidative stress and inflammation. The modification of Keap1 leads to the separation of Nrf2 from Keap1 and Nrf2 accumulation, nuclear translocation, and binding to AREs, followed by the activation of several cytoprotective genes [44, 45]. Several researches have proven that the boost of AMPK or Nrf2 signaling offers effective protection against H_2_O_2_-elicited ROS generation and oxidative stress in chondrocytes. Yang et al. found that several natural ingredient-derived antioxidants exerted chondroprotective effects against H_2_O_2_-elicited oxidative stress via the Nrf2 pathway [46]. Kim et al. found that boost of the Nrf2 pathway in human chondrocytes inhibited H_2_O_2_-induced cell damage [47].

In this study, the results indicated that luteolin led to the stabilization, nuclear translocation, and boost of Nrf2 in primary murine chondrocytes, followed by upregulated expression of Nrf2-ARE-dependent detoxifying enzymes and antioxidant genes, including HO-1, NQO1, and GCLC. Furthermore, luteolin-induced activation of Nrf2 signaling was dose-dependent. These results indicated that luteolin could potently activate the Nrf2 signaling pathway in chondrocytes. Besides, AMPK was activated by luteolin treatment in primary murine chondrocytes, as evidenced by increased AMPK*α*1 phosphorylation. Then, we confirmed that luteolin-induced activation of both the AMPK and Nrf2 pathways protected chondrocytes from H_2_O_2_. Conversely, silencing AMPK or Nrf2 with shRNA or KO reversed luteolin-mediated effects on cell death and apoptosis. Notably, luteolin-induced protection against H_2_O_2_ was almost completely abolished by Nrf2 or AMPK silencing via shRNA or KO in chondrocytes. Taken together, the findings demonstrated that both AMPK and Nrf2 cascade activation mediated luteolin-induced protection against H_2_O_2_ in chondrocytes.

Interestingly, emerging evidence has revealed that AMPK could serve as a key upstream target of the Nrf2 cascade. For example, a recent study showed that AMPK activation improved inflammation and redox imbalance by mediating Nrf2 signaling [16]. Another study revealed that the neuroprotective effect of Nrf2 activation was dependent on the AMPK pathway [17]. Therefore, the interplay between the AMPK and Nrf2 signaling networks in chondrocytes was further clarified. In this study, AMPK silencing or depletion with shRNA or KO completely abolished luteolin-mediated Nrf2 activation. Intriguingly, luteolin-induced AMPK activation, as indicated by increased AMPK*α*1 phosphorylation, was not affected by Nrf2 silencing or depletion. These results indicated that luteolin showed the potential to protect chondrocytes against oxidative injury caused by H_2_O_2_ by activating the AMPK pathway, subsequently resulting in an increase in the downstream Nrf2 cascade.

Finally, we further explored the effects of luteolin in a DMM-induced mouse model, which is widely used for *in vivo* analysis of OA [31]. Histological analysis by HE and safranin O staining, as well as quantitative analysis by OARSI scores, indicated that treatment with luteolin inhibited OA progression, as by less cartilage erosion, chondrocyte loss, and smaller OARSI scores than in DMM mice. Notably, the *in vivo* results also confirmed that luteolin could improve ECM homeostasis, as indicated by decreased MMP13 and increased Nrf2 expression, which was consistent with our *in vitro* results.

There are several limitations that should be put forward. First, the effect of luteolin on OA was based on mouse models instead of human beings. And its effect on mouse kidney and liver was not explored. Besides, the ideal dose, timing, duration of management, and long-term effects by future studies would provide a better understanding of its therapeutic value.

## 5. Conclusion

**Figure 9:**
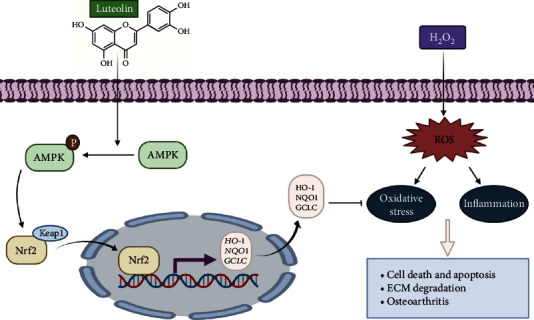
Schematic of the chondroprotective effect of luteolin via the AMPK/Nrf2 pathway. Luteolin protected chondrocytes against H_2_O_2_-induced oxidative stress and inflammation by increasing levels of phosphorylated AMPK and activating Nrf2, which translocates into the nucleus to increase the transcription and expression of Nrf2-target genes, such as HO-1, NQO1, and GCLC.

We demonstrated that luteolin exerted cytoprotective effects against oxidative injury, the inflammatory response, and ECM degradation in primary murine chondrocytes via activating the AMPK/Nrf2 signaling pathway and that AMPK served as a positive upstream regulator of Nrf2. Furthermore, oral administration of luteolin attenuated OA progression in a DMM-induced mouse OA model (Figure 9). Therefore, our findings suggested that luteolin might serve as a novel and effective treatment for OA and provided a new research direction for clinical OA therapies.

## Data Availability

The data used to support the findings of this study are included within the article.
